# Global Proteomic Analysis of Lysine Malonylation in *Toxoplasma gondii*

**DOI:** 10.3389/fmicb.2020.00776

**Published:** 2020-04-28

**Authors:** Lan-Bi Nie, Qin-Li Liang, Rui Du, Hany M. Elsheikha, Nai-Jian Han, Fa-Cai Li, Xing-Quan Zhu

**Affiliations:** ^1^College of Animal Science and Technology, Jilin Agricultural University, Changchun, China; ^2^State Key Laboratory of Veterinary Etiological Biology, Key Laboratory of Veterinary Parasitology of Gansu Province, Lanzhou Veterinary Research Institute, Chinese Academy of Agricultural Sciences, Lanzhou, China; ^3^Faculty of Medicine and Health Sciences, School of Veterinary Medicine and Science, University of Nottingham, Sutton Bonington Campus, Loughborough, United Kingdom; ^4^Jingjie PTM Biolabs (Hangzhou) Co. Ltd., Hangzhou, China

**Keywords:** *Toxoplasma gondii*, toxoplasmosis, post-translational modifications, lysine malonylation, malonylome

## Abstract

Lysine malonylation (Kmal) is a new post-translational modification (PTM), which has been reported in several prokaryotic and eukaryotic species. Although Kmal can regulate many and diverse biological processes in various organisms, knowledge about this important PTM in the apicomplexan parasite *Toxoplasma gondii* is limited. In this study, we performed the first global profiling of malonylated proteins in *T. gondii* tachyzoites using affinity enrichment and Liquid chromatography-tandem mass spectrometry (LC-MS/MS) analysis. Three experiments performed in tandem revealed 294, 345, 352 Kmal sites on 203, 236, 230 malonylated proteins, respectively. Computational analysis showed the identified malonylated proteins to be localized in various subcellular compartments and involved in many cellular functions, particularly mitochondrial function. Additionally, one conserved Kmal motif with a strong bias for cysteine was detected. Taken together, these findings provide the first report of Kmal profile in *T. gondii* and should be an important resource for studying the physiological roles of Kmal in this parasite.

## Introduction

Toxoplasmosis, caused by the protozoan parasite *Toxoplasma gondii*, is estimated to affect approximately one-third of the world population (Montoya and Liesenfeld, 2004). This parasite has the ability to infect almost all mammalian and avian species (Webster, 2010; Robert-Gangneux and Darde, 2012; Liu et al., 2015). The life cycle of *T. gondii* involves definitive host (members of the cat family; Felidae) and intermediate (mammals, including humans) host. During its development, the parasite progresses through three main morphological stages, including one replicative stage (tachyzoite), which is associated with the acute phase of infection, the dormant stage (bradyzoites-containing tissue cyst), which is associated with latent form of infection, and the environmentally resistant oocyst stage. The life cycle of *T. gondii* includes asexual reproduction, which involves the formation of tachyzoites and bradyzoites-containing cysts in the intermediate host and sexual reproduction which involves the formation of oocysts in the feline intestinal epithelium. In order to adapt to different environments and survive inside various tissues within different hosts, the parasite tightly regulates its metabolic and protein functions at the post-translational level (Xiao et al., 2010; Dubey et al., 2017).

Lysine post-translational modifications (PTMs), such as acetylation (Choudhary et al., 2009), methylation (Peng et al., 2011), succinylation (Lin et al., 2012; Hirschey and Zhao, 2015), and ubiquitination (Hershko and Ciechanover, 1998), play key roles in broadening the functional diversity of proteins and impact significantly on the regulation of protein functions in prokaryotic and eukaryotic organisms (Lin et al., 2012; Hirschey and Zhao, 2015). Lysine succinylation has been investigated in *T. gondii*, where it was found to be involved in a broad range of cellular functions (Li et al., 2014). Also, lysine acetylation has been studied in three *T. gondii* strains belonging to three different genotypes and the level of acetylation was found to correlate with the parasite strain virulence and has been found widespread on proteins of diverse functions in *T. gondii* (Jeffers and Sullivan, 2012; Wang et al., 2019).

Post-translational modification of proteins via lysine malonylation (Kmal) has been reported across many metabolic pathways, such as fatty acid synthesis and oxidation (Hirschey and Zhao, 2015), mitochondrial respiration (Xie et al., 2012), glycolysis (Peng et al., 2011; Lin et al., 2012), and modification of histones (Hershko and Ciechanover, 1998). Lysine malonylation was firstly observed in mammalian cells and bacterial cells (Lin et al., 2012). Since then, there have been growing interests in exploring the regulatory roles of Kmal in various microbial species, such as *Escherichia coli* (Qian et al., 2016), *Cyanobacteria* (Ma et al., 2017), and *Saccharopolyspora erythraea* (Xu et al., 2016). Although Kmal can regulate many crucial and diverse cellular processes (He et al., 2012), its existence and function in *T. gondii* remain unknown.

In the present study, we characterized malonylated proteins in *T. gondii* tachyzoites using Liquid chromatography-tandem mass spectrometry (LC-MS/MS) coupled with sensitive immune-affinity purification. Three parallel experiments were performed, which identified 294, 345, 352 Kmal sites on 203, 236, 230 proteins, respectively. Functional analyses showed predominant presence of malonylated proteins in metabolic processes, such as glycolysis/gluconeogenesis, aminoacyl-tRNA biosynthesis, pentose phosphate pathway, and fatty acid biosynthesis. To our knowledge, this study is the first to characterize protein malonylation in *T. gondii*. Our data lay the foundation for future investigations into the biological functions of malonylated proteins in *T. gondii* and in the context of host–parasite interaction.

## Materials and Methods

### Parasite Culture

Tachyzoites of *T. gondii* RH strain were maintained by serial passage in human foreskin fibroblast (HFF) monolayers, which were grown in Dulbecco’s modified Eagle’s medium (DMEM, Gibco, United States) supplemented with 10% fetal calf serum (FBS, Gibco, United States), 100 U/ml antibiotics (penicillin–streptomycin solution). *T. gondii* tachyzoites and HFF monolayers were cultivated in a 5% CO_2_ humidified incubator at 37°C. The tachyzoites and cell debris were harvested when the infected HFF monolayer was lysed. The mixture was washed several times with phosphate buffered saline (PBS) and passed through a 25 gauge needle. Then, the parasites were filtrated using a 3 μm membrane filters (Millipore) in order to remove the cell debris, and stored at −80°C until use.

### Protein Extraction

The parasite pellets were sonicated with 12 short bursts of 10 s, followed by intervals of 10 s on ice for three times in lysis buffer [8 M urea, 1% Protease inhibitor cocktail and for PTM experiments, deacetylase inhibitors were also added to the lysis buffer, e.g., 3 μM trichostatin A (TSA) and 50 mM nicotinamide (NAM)], and the remaining debris was removed by centrifugation at 12,000 *g* for 10 min at 4°C. Finally, the pellet was discarded, and the protein concentration was examined with BCA kit.

### Western Blotting Analysis

Proteins (20 μg) of tachyzoites were electrophoresed on 12% SDS-PAGE and transferred to PVDF membrane. TBST buffer (25 mM Tris–HCl, 150 mM NaCl, 0.1% Tween20, pH 8.0) with 5% BSA was used to block the membrane for 60 min. Then, the membrane was incubated with anti-malonyllysine antibody (1:500, catalog no. PTM-901; PTM Biolabs, Hangzhou, China) in TBST with 2.5% BSA overnight at 4°C. The membrane was washed three times with TBST, followed by incubation with horseradish-peroxidase-conjugated Goat anti-Rabbit IgG (1:5000; Thermo) for 60 min at room temperature. After washing the membrane three times, an ECL kit was used for protein visualization.

### Trypsin Digestion

The protein solution was reduced with 5 mM dithiothreitol (DTT) for 30 min at 56°C and then alkylated with 11 mM iodoacetamide (IAA) for 15 min at 25°C away from light. The proteins were diluted by adding 100 mM NH_4_CO_3_ until the urea concentration became <2 M. Finally, trypsin was added at 1:50 trypsin-to-protein mass ratio for the first digestion overnight and 1:100 trypsin-to-protein mass ratio for a second 4 h-digestion.

### HPLC Fractionation

The tryptic peptides were fractionated by high pH reverse-phase HPLC using Thermo Betasil C18 column (5 μm particles, 10 mm ID, 250 mm length). The peptides were separated using a gradient of 8–32% acetonitrile (pH 9.0) over 60 min into 60 fractions. Thereafter, the separated peptides were combined into four fractions as described previously (Liu et al., 2016).

### Affinity Enrichment

The tryptic peptides dissolved in NETN buffer (100 mM NaCl, 1 mM EDTA, 50 mM Tris–HCl, 0.5% NP-40, pH 8.0) were incubated with pre-washed anti-malonyl-K antibody beads (PTM Biolabs, Hangzhou, China) at 4°C overnight with gentle shaking (Zhang et al., 2016). Then, the beads were washed four times with NETN buffer and twice with H_2_O. The bound peptides were eluted from the beads with 0.1% trifluoroacetic acid (TFA; Sigma-Aldrich, Saint Louis, United States) and were cleaned using C18 ZipTips (Merck Millipore, Billerica, United States).

### Liquid Chromatography-Tandem Mass Spectrometry Analysis

The tryptic peptides were dissolved in 0.1% formic acid (solvent A), directly loaded onto a reverse-phase analytical column (15-cm length, 75 μm i.d.) at a constant flow rate of 700 nL/min on an EASY-nLC 1000 UPLC system. The gradient included an increase from 6–23% solvent B (0.1% formic acid in 98% acetonitrile) over 26 min, followed by 8 min from 23–35%, and reaching to 80% in 3 min and holding at 80% for the last 3 min. The peptides were subjected to a nanospray ionization (NSI) source followed by tandem mass spectrometry (MS/MS) in Q Exactive^TM^ Plus (Thermo) coupled online to an ultra-performance liquid chromatography (UPLC). The *m/z* scan range was 350–1800 for full scan, and intact peptides were detected in the Orbitrap at a resolution of 70,000. A data-dependent procedure was performed, selecting the 20 most intense ions for MS/MS with 30 s dynamic exclusion at a resolution of 17,500.

### Database Search

The MS/MS data were processed using MaxQuant search engine (v.1.5.2.8). Tandem mass spectra were searched against Uniprot *T. gondii* database (ToxoDB 46, 8322 sequences downloaded on March 18, 2020) concatenated with reverse decoy database. Trypsin/P was specified as cleavage enzyme allowing up to 4 missing cleavages. The mass tolerance for fragment ions was set as 0.02 Da. Cysteine carbamidomethylation was specified as fixed modification. Methionine oxidation, and Kmal were specified as variable modifications. False discovery rate (FDR) was adjusted to <1% and minimum score for modified peptides was set >40. All other parameters in MaxQuant were set to default values. The site localization probability was set as >0.75. Label-free quantification was performed using the MaxQuant label free quantification (LFQ) algorithm (Cox et al., 2014), by comparing the abundance of the same peptides across runs, with both ion intensities and spectral counts used for this purpose.

### Bioinformatic Analysis

Subcellular localization of all malonylated proteins identified in three separate experiments was predicted using WOLF PSORT^1^. For Motif analysis, the sequence models contained amino acids at specific positions of the malonyl-21-mers (10 amino acids upstream and downstream of the Kmal sites) in all protein sequences were predicted by MoMo software (motif-x algorithm). The *T. gondii* protein sequences from the UniProt database was used as background database parameter, and other parameters were set as default.

GO annotation was performed against UniProt-GOA database^2^. Firstly, the identified protein identifiers (IDs) were converted to UniProt IDs and then mapped to GO IDs. If the identified malonylated proteins were not annotated by UniProt-GOA database, the InterProScan software were used to annotate protein’s GO function based on protein sequence alignment method. Also, an online service tool, KEGG Automatic Annotation Server (KAAS)^3^, was used to annotate protein’s KEGG database description. Then, the annotation result was mapped to the KEGG pathway database using KEGG mapper. The protein domains were annotated by using InterProScan software and the InterPro domain database^4^ based on protein sequence alignment. A two-tailed Fisher’s exact test was employed for the GO/KEGG/Domain enrichment analysis of the differentially malonylated proteins against all identified proteins. The terms with *P* values <0.05 were considered significant.

All differentially abundant malonylated proteins were searched against the Search Tool for Recurring Instances of Neighboring Genes (STRING) database^5^ for Protein–Protein interaction (PPI). The interaction score was set with high confidence (≧0.7), and all other parameters were set as default. The PPI network was visualized by Cytoscape (version 3.5.0).

## Results And Discussion

Our study provides the first characterization of Kmal, a newly discovered type of lysine acylation, in *T. gondii*. We have identified multiple malonylated proteins with a wide range of functions, such as metabolism and immune response, as discussed below.

### Global Detection of Kmal Sites on *T. gondii* Proteins

**FIGURE 1:**
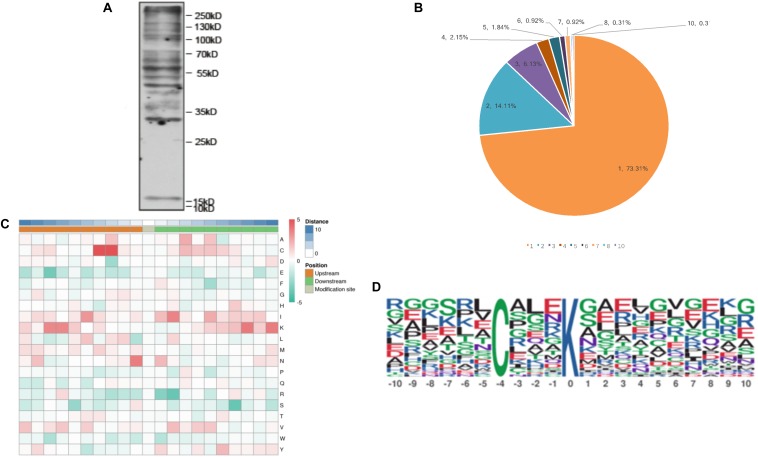
The properties of the lysine malonylation peptides in *Toxoplasma gondii*. **(A)** Western blot analysis of tachyzoite lysate probed with anti-malonylation antibodies. **(B)** The number of Kmal sites for the malonylated protein detected in *T. gondii*. **(C)** Heat map showing different types of amino acids at positions −10 to +10 from the malonylated lysine residue. Red and green color denotes high and low frequency, respectively. **(D)** Sequence motif logos showing the Kmal sites detected in proteins and the position-specific amino acids’ composition surrounding the Kmal sites.

To detect the abundance of malonylated proteins in *T. gondii* tachyzoite extracts, western blotting was performed prior to the proteomic experiments (Figure 1A). The global malonylome analysis of *T. gondii* was performed using affinity enrichment followed by high-resolution LC-MS/MS. Three parallel experiments were performed, which identified 294 Kmal sites on 203 proteins in Exp.1 (Supplementary Table S1), 345 Kmal sites on 236 proteins in Exp.2 (Supplementary Table S2), and 352 Kmal sites on 230 proteins in Exp.3 (Supplementary Table S3). A total of 160 proteins were overlapped between Exp.1 and Exp.2, 151 proteins were overlapped between Exp.1 and Exp.3, 170 proteins were overlapped between Exp.2 and Exp.3, and 138 proteins were overlapped among these three experiments. The low number of overlapped proteins may be attributed to the low incidence of this PTM and the resolution of the methods used, resulting in less repetitions. Overall, 506 Kmal sites across 326 proteins were detected. The number of Kmal proteins in *T. gondii* was higher than that reported in common wheat, *S. erythraea* (Xu et al., 2016; Liu et al., 2018), but was lower than that detected in *E. coli* (Qian et al., 2016), rhizobacterium *Bacillus amyloliquefaciens* FZB42 (Fan et al., 2017) and mammals (Colak et al., 2015), however was close to the number of malonylated proteins identified in *Cyanobacteria* (Ma et al., 2017). As shown in Figure 1B, a total of 326 proteins were modified/malonylated at more than two lysine residues. For example about 73% (239 out of 326) of the identified malonylated proteins were modified at one lysine residue, 14% (46 out of 326) were modified at two lysine residues, and 13% (41 out of 326) were modified at ≥ two lysine residues. The finding that 27% of the malonylated proteins were modified at multiple lysine residues/sites is in agreement with the results detected in wheat and *S. erythraea* (Xu et al., 2016; Liu et al., 2018), indicating some conservation in the level of protein malonylation between *T. gondii* and other organisms, such as bacteria and plants, however the level and site of malonylation may vary among different organisms.

Interestingly, *T. gondii* Elongation Factor 1-Alpha (TgEF-1α) was the largest malonylated protein with 10 malonylated sites, which seems to have functions related to cell motility, protein turnover, cell growth, and signal transduction (Ridgley et al., 1996), DNA replication and repair protein networks (Toueille et al., 2007), suggesting an important, conserved role for Kmal in the activities of this protein. The second highly malonylated protein was the eukaryotic mitochondrial porins which plays a role in transporting tRNAs and apoptosis (Flinner et al., 2013). *T. gondii* 14-3-3 protein had six Kmal sites, this protein is found in the parasitophorous vacuole and the excreted/secreted parasite antigens. *T. gondii* 14-3-3 protein has been suggested to play a role in the modulation of the migratory properties of host cells (Weidner et al., 2016) and host immunity (Assossou et al., 2004). In the present study, five Kmal sites were identified in heat shock protein 70 (Hsp70). A previous study showed that the heat shock protein was the most widely succinylated protein, which can be succinylated on up to 17 independent lysine residues (Li et al., 2014), and the heat shock protein can also be ubiquitinated (Salmon de Monerri et al., 2015). The heat shock protein was shown to play a critical role in modulating host immune response during *Plasmodium falciparum* infection (Pooe et al., 2017) and was also associated with protective immunity against *T. gondii* (Kikumura et al., 2010). These results suggest that Kmal participates in the regulation of various biological processes of different cellular components and influences several biological functions in *T. gondii*.

### Motifs of Malonylated Peptides

To further examine the properties of Kmal sites in *T. gondii*, the flanking sequences from ten amino acids upstream to ten amino acids downstream of the modified site were studied using the MoMo software and hierarchical cluster analysis. As shown in Figure 1C, the frequency of cysteine (C) residue at the position −4 to −3 was highest, the positively charged lysine (K) residue was enriched at the positions −10 to −6 and +10 to +5, whereas the negatively charged residues glutamate (E) and phenylalanine (F) were not observed, this result agree with Kmal reported in common wheat (Liu et al., 2018). Previous study showed that G residues and E residues are highly enriched surrounding the acetylated lysine, while G is overrepresented in the positions on the N-terminal side (Jeffers and Sullivan, 2012). Therefore, the additional characteristics of the surrounding amino acids are not obvious from the motifs, except in some specific positions.

One motif, C (X_3_) Kmal (Kmal indicates the lysine malonylation site, and X represents a random amino acid residue), was obtained (Figure 1D). Except C (X_3_) Kmal, K (X_6_) Kmal, Kmal (X_6_) K, and K (X_5_) Kmal were also detected in *S. erythraea* (Xu et al., 2016). However, the number of Kmal motifs was much lower than lysine acetylation motifs (Li et al., 2016; Lv et al., 2016; Zhang et al., 2016) and succinylation motifs (Pan et al., 2015; Jin and Wu, 2016). The sequence logos show a strong bias for cysteine (C), which was observed at the −3 and −4 positions (Figure 1C), similar to the Kmal bias for arginine (R) detected in the common wheat (Liu et al., 2018).

### Primary GO Enrichment Analysis of the Malonylated Proteins

**FIGURE 2:**
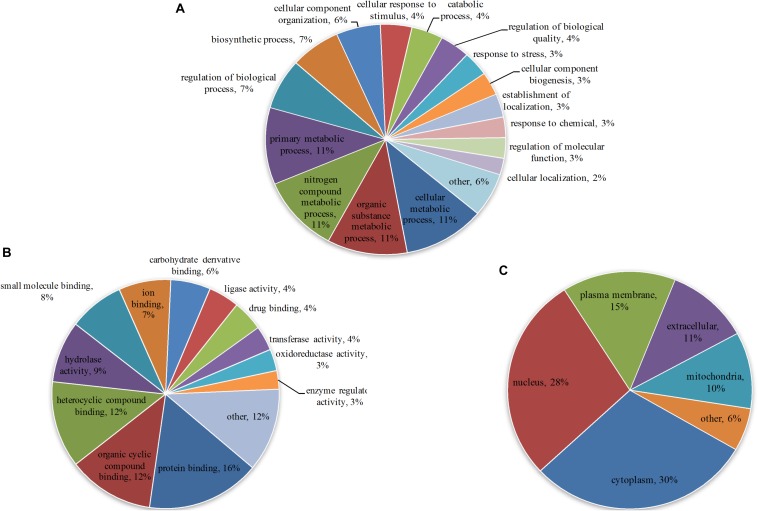
Gene Ontology (GO) functional annotation of the identified malonylated proteins in *Toxoplasma gondii*, including categories: **(A)** Biological process, **(B)** molecular function and **(C)** the GO annotation of the subcellular locations of the malonylated proteins, including the percentages of malonylated proteins involved in specific GO terms in relation to the total malonylated proteins.

To understand the primary function of malonylated proteins, enriched GO terms at 2nd level in the three GO categories: biological process (BP), cellular component (CC) and molecular function (MF) were identified (Supplementary Table S4). In terms of BP, 57, 56, 56 and 56 malonylated proteins were enriched in cellular metabolic process, organic substance metabolic process, nitrogen compound metabolic process and primary metabolic process, accounting for 11% of all identified malonylated proteins in *T. gondii*, respectively (Figure 2A). Regarding the MF category, 16, 12, and 12% of the malonylated proteins were related to protein binding, organic cyclic compound binding and heterocyclic compound binding, respectively (Figure 2B). Also, we used WoLF PSORT to further predict the subcellular locations of the identified proteins (Horton et al., 2007). As shown in Figure 2C, most of the malonylated proteins in *T. gondii* were located in the cytoplasm (30%) and nucleus (28%), the rest of malonylated proteins were predicted to be located in plasma membrane (15%) extracellular (11%) and mitochondria (10%) (Figure 2C).

### Advanced Functional Enrichment of Malonylated Proteins

**FIGURE 3:**
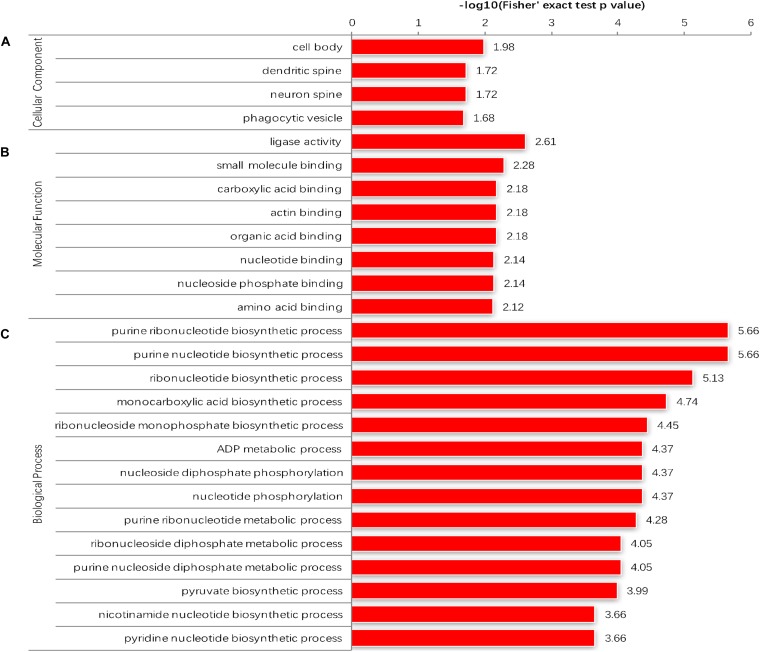
Enrichment analysis of the malonylated proteins in *Toxoplasma gondii* according to the categories of **(A)** cellular component. **(B)** Molecular function. **(C)** Biological process.

**FIGURE 4:**
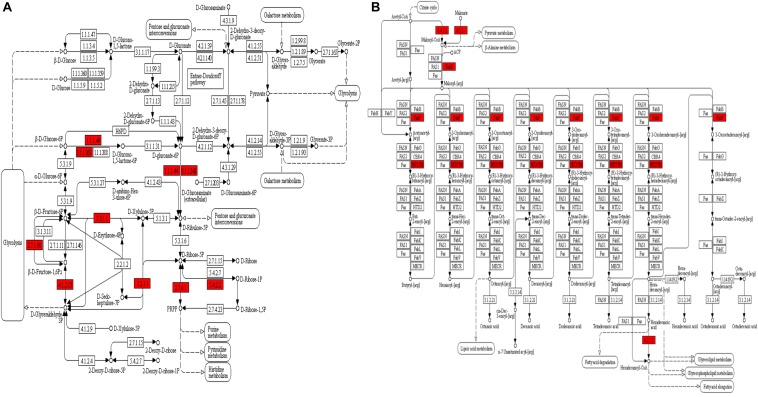
KEGG pathway enrichment analysis of the malonylated proteins in *Toxoplasma gondii*. **(A)** Alterations in the pentose phosphate pathway with significantly enriched modified proteins showing inside red boxes. **(B)** Alterations in the fatty acid biosynthesis pathway with significantly enriched modified proteins are shown inside red boxes.

**FIGURE 5:**
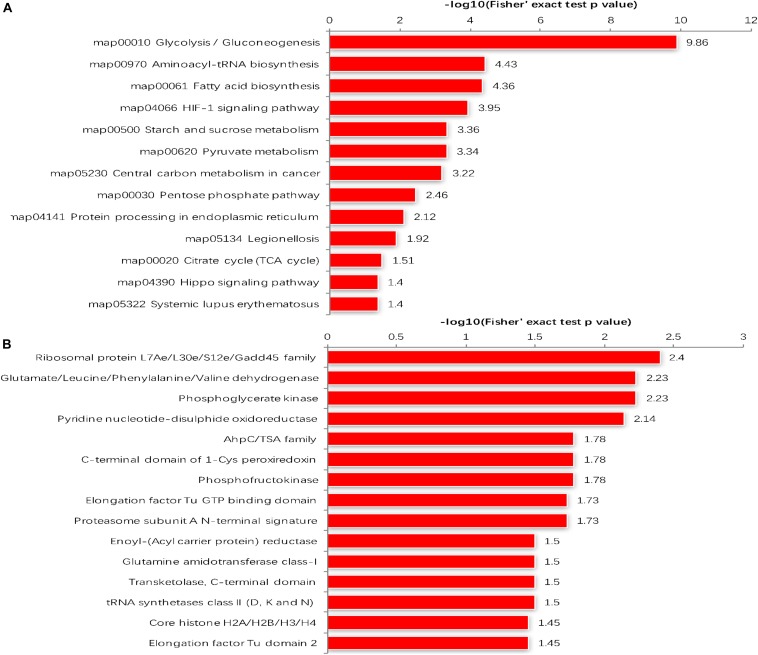
**(A)** KEGG pathways of malonylated proteins. **(B)** Protein domain enrichment analysis.

The GO enrichment analysis and Kyoto Encyclopedia of Genes and Genomes (KEGG) pathway enrichment analysis were performed in *T. gondii*, as shown in Supplementary Table S4. Based on the GO enrichment results of the CC category, malonylated proteins were significantly located in the cell body (Figure 3A). In terms of MF, the most significantly enriched malonylated proteins were related to ligase activity and small molecule binding (Figure 3B). Regarding BP, malonylated proteins were mainly implicated in purine metabolism and monocarboxylic acid biosynthetic processes processes (Figure 3C). Interestingly, seven malonylated proteins in *T. gondii* belonged to the pentose phosphate pathway (Figure 4A). A highly proliferative organism such as *T. gondii* is an avid auxotrophic for many metabolites and must consume these, and carbon sources (e.g., glucose) from the host cell to sustain its growth and proliferation. The pentose phosphate pathway along with glycolysis and mitochondrial respiration are used by the intracellular tachyzoites of *T. gondii* to generate energy and essential anabolic precursors from glucose (MacRae et al., 2012). In addition, eight malonylated proteins in *T. gondii* belonged to the fatty acid biosynthesis (Figure 4B). These are involved in the growth, development, and reproduction of *T. gondii* (Fu et al., 2019). Malonyl-CoA can serve as a substrate of fatty acid synthesis and as an inhibitor of fatty acid oxidation (Foster, 2012). Thus, malonylation might indirectly regulate these important processes in *T. gondii*. KEGG pathway analysis showed that most malonylated proteins are enriched in glycolysis/gluconeogenesis, aminoacyl-tRNA biosynthesis, fatty acid biosynthesis (Figure 5A). Furthermore, enriched protein domain analysis showed that malonylated proteins are significantly enriched in ribosomal protein L7Ae/L30e/S12e/Gadd45 family, glutamate/Leucine/Phenylalanine/Valine dehydrogenase, phosphoglycerate kinase and pyridine nucleotide-disulfide oxidoreductase were significantly enriched in the malonylated proteins (Figure 5B).

**FIGURE 6:**
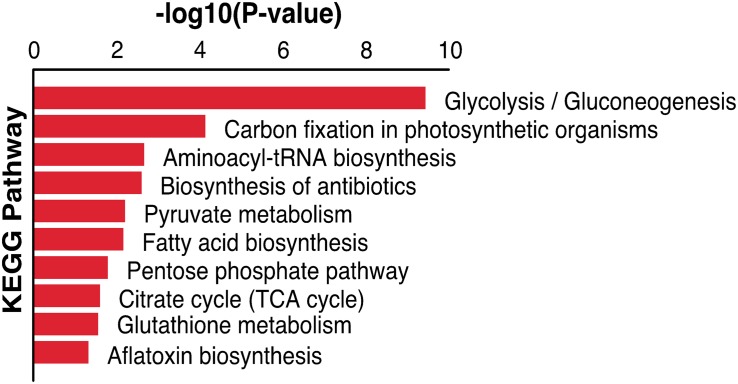
KEGG pathway enrichment analysis of both malonylated and acetylated proteins in *Toxoplasma gondii.*

The malonylated protein fructose-bisphosphate aldolase, which plays a central role in glycolysis and gluconeogenesis pathways, has been considered as a potential target for drug development against pathogenic bacteria (Ziveri et al., 2017). Another malonylated protein (leucyl aminopeptidase; LAP) is a functional aminopeptidase in the cytoplasm of *T. gondii* (Jia et al., 2010). Lactate dehydrogenase (LDH1) was also identified as a malonylated protein, and has been previously shown to catalyze the conversion of pyruvate and lactate in anaerobic growth conditions and used for energy supply (Abdelbaset et al., 2017). *T. gondii* expresses two different lactate dehydrogenase enzyme genes, *LDH*1 is only expressed in the tachyzoite stage whereas *LDH*2 is preferentially expressed in the bradyzoite stage. Given the important role of this enzyme in the parasite virulence, phenotypic differentiation, and latent infection, the potential of using T. gondii LDH mutant strains as vaccine candidates has been suggested (Abdelbaset et al., 2017). Five Kmal sites were identified in glyceraldehyde-3-phosphate dehydrogenase (GAPDH) in the present study, this protein is also succinylated and ubiquitinated in *T. gondii*, and the glyceraldehyde-3-phosphate dehydrogenase I (GAPDH I) was found in the parasite cytoplasm, succinylated on two lysines (Li et al., 2014; Salmon de Monerri et al., 2015). Previous reports showed that phosphorylation and glycation of GAPDH are involved in the regulation of (PPI) and intracellular localization of the enzyme, and that the aggregation of GAPDH can be affected by all types of PTMs (Muronetz et al., 2016). Lysine 213 of GAPDH can be malonylated in macrophages following lipopolysaccharide (LPS) stimulation, which causes its dissociation from several inflammatory mRNAs, promoting translation (Galván-Peña et al., 2019), implying that Kmal has important roles in these processes. A total of 83 malonylated proteins identified in the present study had also been identified as acetylated proteins in a previous study (Jeffers and Sullivan, 2012). KEGG pathway analysis showed that these 83 proteins were mainly enriched in glycolysis/gluconeogenesis, carbon fixation in photosynthetic organisms, aminoacyl-tRNA biosynthesis and biosynthesis of antibiotics (Figure 6), which indicate that malonylation and acetylation may have similarities or synergistic effects on some functions and mechanisms. Further studies are warranted to elucidate the relationship between malonylation and acetylation in *T. gondii.*

### PPI Network of the Malonylated Proteins in *T. gondii*

**FIGURE 7:**
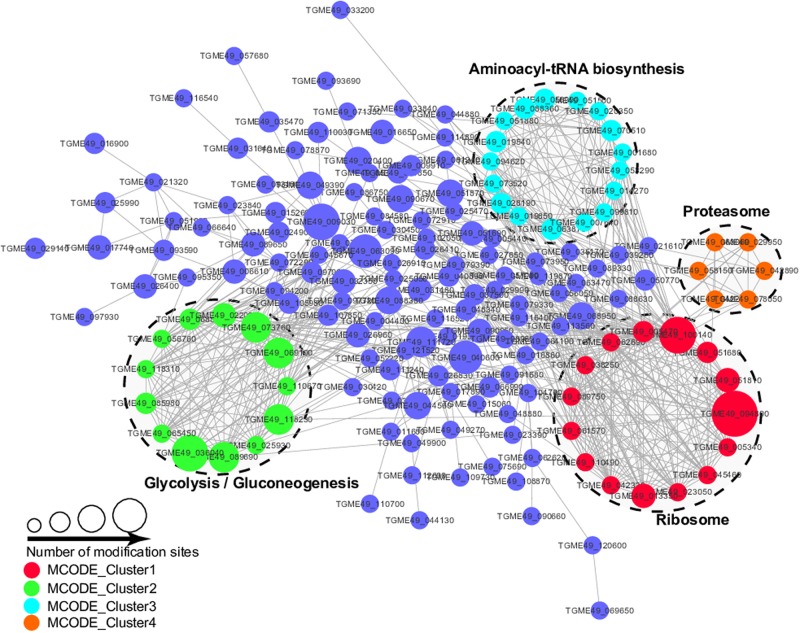
Protein–Protein interaction networks of malonylated proteins in *Toxoplasma gondii*. Nodes represent the malonylated proteins and edges represent interactors between malonylated proteins. The color of the edges denotes the combined score of interactors.

The PPI analysis was performed by searching the STRING database and PPI networks were visualized using Cytoscape software in order to further identify the major biological processed affected by Kmal in *T. gondii* (Shannon et al., 2003; Figure 7, Supplementary Table S5). Using MCODE (Minimal Common Oncology Data Elements), a number of highly associated subnetworks of Kmal proteins were identified, including glycolysis/gluconeogenesis, ribosome, proteasome and aminoacyl-tRNA biosynthesis processes, which are in agreement with the KEGG pathway enrichment analysis, suggesting that these processes play important roles in shaping the proteomic landscape of *T. gondii*.

## Conclusion

In this study, we described the first malonylome profile in *T. gondii* using mass spectrometry in combination with immune-affinity purification. Over 300 proteins were found to be malonylated and were localized in different cellular compartments. These malonylated proteins were associated with diverse biological and metabolic processes, suggesting that Kmal is involved in the regulation of *T. gondii* physiology. We also found that some important proteins related to *T. gondii* invasion, emergence and virulence were malonylated, which suggest that Kmal plays an important role in the regulation of functions of these critical proteins of *T. gondii*. Our findings will serve as a useful resource for further investigation of the functions of Kmal, especially in the context of parasite interaction with the host cells. Further studies should focus on the experimental verification of the functions of these malonylated proteins to reveal the exact roles of malonylation in *T. gondii*. Analysis of the extent of differences and similarities in Kmal between different developmental stages (bradyzoites, tachyzoites and oocysts) and between distinct genotypes of *T. gondii* is warranted, which in turn will facilitate the elucidation of mechanisms underlying the phenotypic transformation and varying virulence of *T. gondii*.

## Data Availability Statement

All the mass spectrometry data have been submitted to the ProteomeXchange Consortium with the identifier PXD015809.

## Author Contributions

RD, F-CL, and X-QZ conceived and designed the research. L-BN performed the research, analyzed the data and drafted the manuscript. Q-LL, HE, and N-JH contributed materials, reagents, and analysis tools. HE, F-CL, RD, and X-QZ critically revised the manuscript. All authors reviewed and approved the final version of the manuscript.

## Conflict of Interest

N-JH was employed by Jingjie PTM Biolabs (Hangzhou) Co., Ltd. The remaining authors declare that the research was conducted in the absence of any commercial or financial relationships that could be construed as a potential conflict of interest.

## Footnotes

1 http://www.genscript.com/wolf-psort.html

2 http://www.ebi.ac.uk/GOA/

3 http://www.genome.jp/kegg/kaas/

4 http://www.ebi.ac.uk/interpro/

5 https://string-db.org/

## Supplementary Material

TABLE S1 The identified Kmal sites in *T. gondii* in Exp.1.

TABLE S2 The identified Kmal sites in *T. gondii* in Exp.2.

TABLE S3 The identified Kmal sites in *T. gondii* in Exp.3.

TABLE S4 Annotations of malonylated proteins including information on Gene Ontology (GO), protein domains, KEGG pathways, and subcellular localization.

TABLE S5 Protein interaction network of the identified malonylated proteins.

## References

[B1] Abdelbaset A. E. Fox B. A. Karram M. H. Abd Ellah M. R. Bzik D. J. Igarashi M. (2017). Lactate dehydrogenase in *Toxoplasma gondii* controls virulence, bradyzoite differentiation, and chronic infection. *PLoS One* 12:e0173745. 10.1371/journal.pone.0173745 28323833 PMC5360243

[B2] Assossou O. Besson F. Rouault J. P. Persat F. Ferrandiz J. Martine M. (2004). Characterization of an excreted/secreted antigen form of 14-3-3 protein in *Toxoplasma gondii* tachyzoites. *FEMS Microbiol. Lett.* 234:25. 10.1016/j.femsle.2004.02.024 15109715

[B3] Choudhary C. Kumar C. Gnad F. Nielsen M. L. Rehman M. Walther T. C. (2009). Lysine acetylation targets protein complexes and co-regulates major cellular functions. *Science* 325 834–840. 10.1126/science 19608861

[B4] Colak G. Pougovkina O. Dai L. Tan M. Brinke H. Huang H. (2015). Proteomic and biochemical studies of lysine malonylation suggest its malonic acidaria-associated regulatory role in mitochondrial function and fatty acid oxidation. *Mol. Cell. Proteomics* 14 3056–3071. 10.1074/mcp.M115.048850 26320211 PMC4638046

[B5] Cox J. Hein M. Y. Luber C. A. Paron I. Nagaraj N. Mann M. (2014). Accurate proteome-wide label-free quantification by delayed normalization and maximal peptide ratio extraction, termed MaxLFQ. *Mol. Cell. Proteomics* 13 2513–2526. 10.1074/mcp.M113.031591 24942700 PMC4159666

[B6] Dubey R. Staker B. L. Foe I. T. Bogyo M. Myler P. J. Ngô H. M. (2017). Gubbels M.J. Membrane skeletal association and post-translational allosteric regulation of *Toxoplasma gondii* GAPDH1. *Mol. Microbiol.* 103 618–634. 10.1111/mmi 27859784 PMC5296235

[B7] Fan B. Li Y. L. Li L. Peng X. J. Bu C. Wu X. Q. (2017). Malonylome analysis of rhizobacterium *Bacillus amyloliquefaciens* FZB42 reveals involvement of lysine malonylation in polyketide synthesis and plant-bacteria interactions. *J. Proteome* 154 1–12. 10.1016/j.jprot.2016.11.022 27939684

[B8] Flinner N. Ellenrieder L. Stiller S. B. Becker T. Schleiff E. Mirus O. (2013). Mdm10 is an ancient eukaryotic porin co-occurring with the ERMES complex. *Biochim. Biophys. Acta* 1833 3314–3325. 10.1016/j.bbamcr.2013.10.006 24135058

[B9] Foster D. W. (2012). Malonyl-CoA: the regulator of fatty acid synthesis and oxidation. *J. Clin. Invest.* 122 1958–1959. 10.1172/jci63967 22833869 PMC3366419

[B10] Fu Y. Cui X. Liu J. Zhang X. Zhang H. Yang C. (2019). Synergistic roles of acyl-CoA binding protein (ACBP1) and sterol carrier protein 2 (SCP2) in *Toxoplasma* lipid metabolism. *Cell. Microbiol.* 21:e12970. 10.1111/cmi.12970 30362657

[B11] Galván-Peña S. Carroll R. G. Newman C. Hinchy E. C. Palsson-McDermott E. Robinson E. K. (2019). Malonylation of GAPDH is an inflammatory signal in macrophages. *Nat. Commun.* 10:338. 10.1038/s41467-018-08187-6 30659183 PMC6338787

[B12] He W. Newman J. C. Wang M. Z. Ho L. Verdin E. (2012). Mitochondrial sirtuins: regulators of protein acylation and metabolism. *Trends Endocrin. Met.* 23 467–476. 10.1016/j.tem.2012.07.004 22902903

[B13] Hershko A. Ciechanover A. (1998). The ubiquitin system. *Annu. Rev. Biochem.* 67 425–479. 10.1146/annurev.biochem.67.1.425 9759494

[B14] Hirschey M. D. Zhao Y. (2015). Metabolic regulation by lysine malonylation, succinylation and glutarylation. *Mol. Cell. Proteomics* 14 2308–2315. 10.1074/mcp.R114.046664 25717114 PMC4563717

[B15] Horton P. Park K. J. Obayashi T. Fujita N. Harada H. Adams-Collier C. J. (2007). WoLF PSORT: protein localization predictor. *Nucleic Acids Res.* 35 W585–W587. 10.1093/nar/gkm259 17517783 PMC1933216

[B16] Jeffers V. Sullivan W. J. (2012). Lysine acetylation is widespread on proteins of diverse function and localization in the protozoan parasite *Toxoplasma gondii*. *Eukaryot. Cell* 11 735–742. 10.1128/EC.00088-12 22544907 PMC3370464

[B17] Jia H. Nishikawa Y. Luo Y. Yamagishi J. Sugimoto C. Xuan X. (2010). Characterization of a leucine aminopeptidase from *Toxoplasma gondii*. *Mol. Biochem. Parasitol.* 170 1–6. 10.1016/j.molbiopara.2009.11.005 19931316

[B18] Jin W. Wu F. (2016). Proteome-wide identification of lysine succinylation in the proteins of tomato (*Solanum lycopersicum*). *PLoS One* 11:e0147586. 10.1371/journal.pone.0147586 26828863 PMC4734689

[B19] Kikumura A. Fang H. Mun H. S. Uemura N. Makino M. Sayama Y. (2010). Protective immunity against lethal anaphylactic reaction in *Toxoplasma gondii*-infected mice by DNA vaccination with T. gondii-derived heat shock protein 70 gene. *Parasitol. Int.* 59 105–111. 10.1016/j.parint.2010.03.006 20346412

[B20] Li D. Lv B. Tan L. Yang Q. Liang W. (2016). Acetylome analysis reveals the involvement of lysine acetylation in diverse biological processes in *Phytophthora sojae*. *Sci. Rep.* 6:29897. 10.1038/srep29897 27412925 PMC4944153

[B21] Li X. Hu X. Wan Y. Xie G. Li X. Chen D. (2014). Systematic identification of the lysine succinylation in the protozoan parasite *Toxoplasma gondii*. *J. Proteome Res.* 13 6087–6095. 10.1021/pr500992r 25377623

[B22] Lin H. Su X. He B. (2012). Protein lysine acylation and cysteine succination by intermediates of energy metabolism. *ACS Chem. Biol.* 7 947–960. 10.1021/cb3001793 22571489 PMC3376250

[B23] Liu J. Wang G. Lin Q. Liang W. Gao Z. Mu P. (2018). Systematic analysis of the lysine malonylome in common wheat. *BMC Genomics* 19:209. 10.1186/s12864-018-4535-y 29558883 PMC5859436

[B24] Liu L. Wang G. Song L. Lv B. Liang W. (2016). Acetylome analysis reveals the involvement of lysine acetylation in biosynthesis of antibiotics in *Bacillus amyloliquefaciens*. *Sci. Rep.* 6:20108. 10.1038/srep20108 26822828 PMC4731788

[B25] Liu Q. Wang Z. D. Huang S. Y. Zhu X. Q. (2015). Diagnosis of toxoplasmosis and typing of *Toxoplasma gondii*. *Parasit. Vectors* 8:292. 10.1186/s13071-015-0902-6 26017718 PMC4451882

[B26] Lv B. Yang Q. Li D. Liang W. Song L. (2016). Proteome-wide analysis of lysine acetylation in the plant pathogen *Botrytis cinerea*. *Sci. Rep.* 6:29313. 10.1038/srep29313 27381557 PMC4933888

[B27] Ma Y. Yang M. Lin X. Liu X. Huang H. Ge F. (2017). Malonylome analysis reveals the involvement of lysine malonylation in metabolism and photosynthesis in cyanobacteria. *J. Proteome Res.* 16 2030–2043. 10.1021/acs.jproteome.7b00017 28365990

[B28] MacRae J. I. Sheiner L. Nahid A. Tonkin C. Striepen B. McConville M. J. (2012). Mitochondrial metabolism of glucose and glutamine is required for intracellular growth of *Toxoplasma gondii*. *Cell Host Microbe* 12 682–692. 10.1016/j.chom.2012.09.013 23159057 PMC3990185

[B29] Montoya J. G. Liesenfeld O. (2004). Toxoplasmosis. *Lancet* 363 1965–1976. 10.1016/S0140-6736(04)16412-X 15194258

[B30] Muronetz V. I. Barinova K. V. Stroylova Y. Y. Semenyuk P. I. Schmalhausen E. V. (2016). Glyceraldehyde-3-phosphate dehydrogenase: aggregation mechanisms and impact on amyloid neurodegenerative diseases. *Int. J. Biol. Macromol.* 100 55–66. 10.1016/j.ijbiomac.2016.05.066 27215901

[B31] Pan J. Chen R. Li C. Li W. Ye Z. (2015). Global analysis of protein lysine succinylation profiles and their overlap with lysine acetylation in the marine bacterium *Vibrio parahemolyticus*. *J. Proteome Res.* 14 4309–4318. 10.1021/acs.jproteome.5b00485 26369940

[B32] Peng C. Lu Z. Xie Z. Cheng Z. Chen Y. Tan M. (2011). The first identification of lysine malonylation substrates and its regulatory enzyme. *Mol. Cell. Proteomics* 10 M111.012658. 10.1074/mcp.M111.012658 21908771 PMC3237090

[B33] Pooe A. Gabriele K. Heine H. Shonhai A. (2017). *Plasmodium falciparum* heat shock protein 70 lacks immune modulatory activity. *Protein Peptide Lett.* 24 503–510. 10.2174/0929866524666170214141909 28201964

[B34] Qian L. Nie L. Chen M. Liu P. Zhu J. Zhai L. (2016). Global profiling of protein lysine malonylation in *Escherichia coli* reveals its role in energy metabolism. *J. Proteome Res.* 15 2060–2071. 10.1021/acs.jproteome.6b00264 27183143

[B35] Ridgley E. L. Xiong Z. H. Kaur K. J. Ruben L. (1996). Genomic organization and expression of elongation factor-1 alpha genes in *Trypanosoma brucei*. *Mol. Biochem. Parasitol.* 79 119–123. 10.1016/0166-6851(96)02639-4 8844680

[B36] Robert-Gangneux F. Darde M. L. (2012). Epidemiology of and diagnostic strategies for toxoplasmosis. *Clin. Microbiol. Rev.* 25 264–296. 10.1128/CMR.05013-11 22491772 PMC3346298

[B37] Salmon de Monerri N. C. Yakubu R. R. Chen A. L. Bradley P. J. Nieves E. Weiss L. M. (2015). The ubiquitin proteome of *Toxoplasma gondii* reveals roles for protein ubiquitination in cell-cycle transitions. *Cell Host Microb.* 18 621–633. 10.1016/j.chom.2015.10.014 26567513 PMC4968887

[B38] Shannon P. Markiel A. Ozier O. Baliga N. S. Wang J. T. Ramage D. (2003). Cytoscape: a software environment for integrated models of biomolecular interaction networks. *Genome Res.* 13 2498–2504. 10.1101/gr.1239303 14597658 PMC403769

[B39] Toueille M. Saint-Jean B. Castroviejo M. Benedetto J. P. (2007). The elongation factor 1A: a novel regulator in the DNA replication/repair protein network in wheat cells? *Plant Physiol. Biochem.* 45 113–118. 10.1016/j.plaphy.2007.01.006 17344053

[B40] Wang Z. X. Hu R. S. Zhou C. X. He J. J. Elsheikha H. Zhu X. Q. (2019). Label-free quantitative acetylome analysis reveals *Toxoplasma gondii* genotype-specific acetylomic signatures. *Microorganisms* 30 7. 10.3390/microorganisms7110510 31671511 PMC6921067

[B41] Webster J. P. (2010). Dubey, j. p. toxoplasmosis of animals and humans. *Parasit. Vectors* 3:112 10.1186/1756-3305-3-112

[B42] Weidner J. M. Kanatani S. Uchtenhagen H. Varas-Godoy M. Schulte T. Engelberg K. (2016). Migratory activation of parasitized dendritic cells by the protozoan *Toxoplasma gondii* 14-3-3 protein. *Cell. Microbiol.* 18 1537–1550. 10.1111/cmi.12595 27018989 PMC5040621

[B43] Xiao H. El Bissati K. Verdier-Pinard P. Burd B. Zhang H. Kim K. (2010). Post-translational modifications to *Toxoplasma gondii* alpha- and beta-tubulins include novel C-terminal methylation. *J. Proteome Res.* 9 359–372. 10.1021/pr900699a 19886702 PMC2813730

[B44] Xie Z. Dai J. Dai L. Tan M. Cheng Z. Wu Y. (2012). Lysine succinylation and lysine malonylation in histones. *Mol. Cell. Proteomics* 11 100–107. 10.1074/mcp.m111.015875 22389435 PMC3418837

[B45] Xu J. Y. Xu Z. Zhou Y. Ye B. C. (2016). Lysine malonylome may affect the central metabolism and erythromycin biosynthesis pathway in *Saccharopolyspora erythraea*. *J. Proteome Res.* 15:1685 10.1021/acs.jproteome.6b00131 27090497

[B46] Zhang Y. Song L. Liang W. Mu P. Wang S. Lin Q. (2016). Comprehensive profiling of lysine acetylproteome analysis reveals diverse functions of lysine acetylation in common wheat. *Sci. Rep.* 6:21069. 10.1038/srep21069 26875666 PMC4753473

[B47] Ziveri J. Tros F. Guerrera I. C. Chhuon C. Audry M. Dupuis M. (2017). The metabolic enzyme fructose-1,6-bisphosphate aldolase acts as a transcriptional regulator in pathogenic *Francisella*. *Nat. Commun.* 8:853. 10.1038/s41467-017-00889-7 29021545 PMC5636795

